# Biosorbent treatment of fluorene using activated carbon derived from the pyrolysis process of date pit wastes

**DOI:** 10.1038/s41598-024-72127-2

**Published:** 2024-09-26

**Authors:** Tarek O. Said, Badriah S. Al-Farhan, Sara A. El-Ghamdi, Nasser Awwad

**Affiliations:** 1 National Institute of Oceanography and Fisheries (NIOF), Cairo, Egypt; 2 Department of Chemistry, College of Science for Girls, King Khalid University, Abha, Saudi Arabia; 3 Department of Chemistry, College of Science, King Khalid University, Alfarah, Abha, Saudi Arabia

**Keywords:** AC, Date pits, FLU, Wastes, Treatment, Biophysics, Environmental sciences, Risk factors, Chemistry

## Abstract

Activated carbon (AC) derived from Date pits (DP) wastes was used as an eco-friendly and effective biosorbent for the removal of fluorene (FLU) from organic wastes. The maximum capacity of DP was 6.71 mg g^−1^, compatible with the Freundlich model. FLU adsorption's chemisorption performance on DP was involved in following a superior linear fit for the pseudo-2nd-kinetic model. The maximum adsorption capacity from the pseudo-2nd order kinetic model fitted with the experimental findings and found to be 3.73 g, 2.62, 1.13, 0.955, 0.749, 0.591, and 0.665 mg g^−1^ at 25, 3, 35, 4, 45, 5 and 55 °C, respectively. The negative value of the spontaneous nature of the adsorption corresponds to the exothermic nature however, + ΔS corresponds to an increase in the degree of freedom for FLU adsorption. The relatively high value of activation energy (Ea) demonstrates that the adsorption of FLU onto DP is classified as chemical adsorption, and found to be 84.8 kJ mol^−1^. Also, the result of XRD shows that the prepared DP was re-used four times without substantially decreasing performance. In addition, it appears that AC prepared from DP is a promising adsorbent with a low cost for removing many organic pollutants.

## Introduction

Because polycyclic aromatic hydrocarbons (PAHs) have extreme toxicity across the ecosystem, long-term measures are desperately needed to accelerate their elimination. It has been shown that physical and/or chemical conventional techniques have significant drawbacks, including technological complexity, expensive costs, and a general lack of acceptance^1^. There have been several promising methods for cleaning up PAH-contaminated environments. Soil cleaning, flushing, thermal desorption, land farming, vapour extraction, excavation and landfilling, bioremediation, and other cutting-edge technologies have all been discussed previously^2^. Due to its broad application, excellent performance, and financial viability, adsorption has demonstrated several benefits that make it one of the most significant purification techniques. It is a high-quality treatment method for the removal of contaminants. Adsorbent processes are classified into two categories based on the type of interactions between the adsorbents: adsorption processes include chemical and physical^3^. Activated carbon (AC) is a porous material with unique chemical and physical properties, and it’s a promising solid carrier that has been widely used in various applications^4^. A lot of research has been done on date pits (DP) to produce AC. Surkatti et al.^5^ focused on the chemical composition, DP availability, and methods for preparing AC from DP. Additionally, the adsorption mechanisms, contaminant removal efficiencies, and characteristics of AC-DP are described. Because AC is a carbonaceous material with a high degree of porosity, a well-developed surface area, and distinct functional groups—all essential for contaminant removal—it is one of the best options for eliminating pollutants from aqueous and atmospheric ecosystems. The straightforward and secure process of pyrolysis, or gasification, of biomass using heat and/or chemicals produces AC. AC needs to be recycled and renewed after usage for resource management and environmental safety. Consequently, AC can protect the environment in two ways: by eliminating pollutants and cleaning the air and water^6^. Livestock, industrial byproducts, and agricultural waste can all be used to make AC. The most widely used commercial sources of activated carbon are bituminous and anthracite charcoal, wood, lignite, peat moss, and coconut^7^. El-Naas et al.^8^ conducted experiments on the batch adsorption of phenol from petroleum refinery wastewater using AC made locally from DP. Adsorption equilibrium and kinetics data were obtained to remove phenol from both synthetically manufactured aqueous phenol solutions and real wastewater from refineries. Several adsorption isotherms and kinetics models were used to match the data. The pseudo-2nd-order model fits the kinetics data best, while the Sips and Langmuir models best fit the equilibrium isotherms. The exothermic nature of the adsorption process was demonstrated by the enthalpy of adsorption. After four regeneration cycles, ethanol was found to be the most effective chemical and thermal method for regenerating saturated AC, with a regeneration efficiency of over 86%. A different study regarding the adsorption of granular activated carbon (GAC) was proposed, which was employed to eliminate NAP, ACY, ACE, FLU, and PHE, from water. The pseudo-1st-order (PFO) model provided a satisfactory explanation for the kinetics of PAH adsorption. Intra-particle diffusion rates into the GAC's mesopores and micropores were found to be higher and slower, respectively, according to the results fitted by the Weber and Morris diffusion models. The molar volumes of the PAHs were inversely correlated with these speeds. The adsorption affinities, which were connected to the PAHs' hydrophobicity, were ascertained using the log Kow values. The effective kinetic data fitting to the PFO model and the anticipated free energy of adsorption from the Dubinin Radushkevich model both demonstrate that the PAHs were physically adsorbed. The Thomas model successfully replicated NAP, ACY, and ACE adsorption in fixed-bed columns containing a combination of GAC 0.5 g and sand 24.5 g^9^. Kumar et al.^10^ investigated the efficaciousness of pyrolysis-assisted potassium hydroxide-induced palm shell-AC (PK-PSAC) and KPSAC in removing PAH from aqueous medium. Both before and after adsorption, the PK-PSAC's SEM and HPLC analyses were examined. The removal of ACE was examined under a range of operational conditions, encompassing temperature, contact time, pH, concentration, and contact time. The highest adsorption capacities were demonstrated by K-PSAC and PK-PSAC, with 96.5 and 132 mg g^−1^, respectively. The isotherm and kinetic studies for PAH adsorption onto PK-PSAC were faithfully portrayed by the pseudo-1st-order and Freundlich models, respectively. The operating parameters for the aqueous sample were found to be optimal at an inlet flow rate of 10–15 mL min^−1^, an AC concentration of 50 mg L^−1^, and 200 mm in fixed-bed column tests. The findings demonstrated that the amount of ACE adsorption decreased with increasing flow conditions and increased with each rise in PAH content. Yoon Nelson and Thomas-Adams models were used to forecast the adsorption column's dynamic behavior. Pyrolysis was found to be a viable alternative technique for increasing the adsorbent limit while still allowing for the effective evacuation of hazardous pollutants in this study. Another study used AC made from banana peel waste to remove all PAHs from the aqueous system economically and effectively. The Brunauer-Emmett-surface Teller's area N_2_ BPAC is over 900 m^2^ g^−1^. This research looked at the effects of the effects of different values of pH, adsorbent dosage, contact time, and temperature on PAH absorption. The primary findings were fitted to Freundlich and Langmuir models, and characteristic values for both adsorption isotherms were derived. The Freundlich model is better suited to the data. Absorption on BPAC has an equilibrium time of 80 min. The equilibrium constants derived from Langmuir adsorption isotherms were used to calculate thermodynamic variables such as S, H, and G. The findings revealed that PAH absorption on BPAC is both automated and endothermic. Using an ethanol and NaOH mixture, the PAHs were quantitatively absorbed from BPAC, demonstrating the reuse of the activated carbon. Consequently, the study suggests an inexpensive, easy-to-use absorbent that effectively removes PAHs from the aqueous system^11^. When it comes to biological treatment, conventional methods are inadequate for managing highly concentrated liquid effluents. Nor El houda et al.^12^ conducted a study to examine the effectiveness of a hybrid adsorption electrocoagulation (AD-EC) process in treating the refinery effluent of Algiers. The purpose of the study was to evaluate the ability of the AD-EC method to eliminate three important contaminants: total suspended solids (TSS), turbidity, and chemical oxygen demand (COD). Aluminum electrodes and AC served as the adsorbents in the EC process. For hybrid AD-EC processes, unfavorable operating parameters were used, such as adsorbent dosage, current density, and electrolysis duration. The solid produced was analyzed using SEM, EDS, and BET. The outcome showed that the hybrid process was effective in eliminating turbidity, TSS, and COD while also saving treatment time. 82.64% of COD, 96.50% of turbidity, and 96.64% of TSS were eliminated as a result of the treatment, all of which were well within Algeria's specified discharge limits. Moreover, these findings support the idea that AD and EC work in concert. Additionally, by improving effluent quality and overall biological treatment efficiency, this hybrid approach has the potential to be a workable tertiary treatment step and a viable ETP solution. Alhusnawy and Alsultani^13^ looked into the development of affordable adsorption methods to remove toluene from water using AC derived from corn biomass. Tested in a continuous fluidized bed column, the AC was created by carbonizing and surface-modifying zinc chloride. The ideal parameters were a bed height of 10 cm, a temperature of 30 °C, and a flow rate of contaminated water of 15 L h^−1^. These values allowed the adsorbents to remove toluene with effectiveness. To evaluate their effect on toluene adsorption efficiency, operational parameters such as temperature 30–40 °C, bed heights 6–10 cm, and flow rates 15–25 L h^−1^ were changed. The analysis, which employed a variety of kinetic models, including Adams-Bohart and Thomas, showed a strong correlation R^2^ > 0.89 with the Thomas model, suggesting a 2nd-order kinetic reaction and Langmuir isotherm behavior. These results show that AC from biomass from corn can be used in adsorption processes to remove toluene from contaminated water. Phosphoric acid was used to chemically activate DP AC, a novel and eco-friendly adsorbent, which was then used as a bio-sorbent for Cd^2+^ adsorption from a simulated polluted solution. The surface morphology and functional groups of raw DP and manufactured AC are identified using a range of characterization techniques. The prepared AC showed the biggest increase in surface area, with adsorption surface area and pore diameter values of 1700 m^2^ g^−1^ and 3.78 nm, respectively^14^. DP AC Cd^2+^ removal was investigated to pH, initial Cd^2+^ concentration, contact time, and operating temperature. At 30 °C, a pH of 6, a contact time of 240 min, and an initial concentration of 100 mg L^−1^, a greater Cd^2+^ adsorption capacity of 48.3 mg g^−1^ was obtained. As the Cd^2+^ concentration dropped and the adsorbate's initial pH rose to 6, the effectiveness of Cd^2+^ adsorption on date pit activated carbon improved.

Lei et al.^15^ successfully prepared a functional composite membrane on seaweed residue (SR) grafted with oxidized graphene (GO). It activated graphene oxide/seaweed residue-zirconium dioxide (GOSRZ) with fluoride removal, uranium extraction, and antibacterial activity through biomimetic mineralization of zirconium dioxide nanoparticles (ZrO_2_ NPs) on SR. The GOSRZ membrane demonstrates extremely effective and targeted fluoride adsorption. Even in the presence of interfering ions, removal efficiency can reach over 99% for fluoride concentrations in water within the 100–400 mg L^−1^ range. The GOSRZ membrane also yields satisfactory extraction rates for uranium. Furthermore, the antibacterial performance studies demonstrate that this composite membrane effectively eliminates Methicillin-resistant *Staphylococcus aureus* (MRSA) and *Escherichia coli* (*E. coli*). The antibacterial activity of the composite membrane is attributed to the destruction of bacterial cell structure, while the high adsorption of F^−^ and U(VI) is ascribed to ionic exchange and coordination interactions. By using microwave pyrolysis to load MgO, Fe_2_O_3_, and Fe_3_O_4_ nanoparticles onto carbon fibre, a magnetic composite adsorbent material (MgO/Fe_2_O_3_/Fe_3_O_4_/CF) was created for the removal of fluoride^16^. The adsorbent was carefully characterized to confirm the metal oxide payload on the carbon foam. The highest adsorption capacity measured was 249.0 mg/g, and the adsorption process in practice is consistent with the pseudo-2nd-order kinetic model and Langmuir isotherm model. Among the complex adsorbents reported in the past, a high adsorption capacity like this is uncommon. The examination of the water chemical equilibrium parameters, EDS, FTIR, XPS, and XRD data also suggested a potential mechanism. The reactivity, adsorption energy between F^−^ and metal oxides, and interaction configurations of F^−^ with hydrated ions were calculated using Density Functional Theory (DFT). The use of AC has demonstrated the efficacy of the adsorption process in treating wastewater dyes, but the production of AC is restricted by the non-renewable and relatively expensive precursor of coal^17^. Date palm residues (DPRs) offer a viable substitute for the precursor of AC because of their large availability, continuous supply, and good physiochemical properties (high oxygen content and fixed carbon). Their study reviews the potential applications of DPRs as AC in adsorbing textile dyes as well as the recent technological advancements used by researchers to produce DPR-based AC. Their review article focuses solely on DPR and not on other biomass waste. Their study presents a background review of date palms, textile dyes, biochar, and AC, followed by the production methods of AC. In addition, Hua et al.^18^ stated that FLU became the primary component in composted sludge material and the possible main limiting factor for PAH contamination for the composted material to be used on agricultural land. Al-Zuhair et al.^19^ used DP as adsorbing agent for transformer oil. They proved that the regenerated oil after adsorption was found to approach acceptable standards. The information above makes it evident that, depending on the situation, AC taken from DP wastes can be effectively used to treat organic pollutants. The present study aimed to investigate the sorption of FLU using DP in an aqueous medium using different analytical tools. The efficiency of the DP to adsorb FLU from organic wastes was studied. Various factors were studied, such as shaking time, concentrations, adsorbent dose, pH, and temperature. Adsorption isotherm models were applied to the results obtained by Langmuir and Freundlich. Batch experiments were carried to find the optimum conditions for removing FLU, selected as an example of the most common PAH pollutant in the environment. In addition, the novelty of the present work is to produce reused AC from DP wastes as an eco-friendly and effective biosorbent for removing FLU from organic wastes at low cost. Reusability experiments using ethanol, sodium hydroxide, acetic acid, hydrochloric acid, and sulfuric acid to deplete the regenerated DP. Because of its ease of use and adaptability, adsorption is frequently regarded as the most practical method of treating water when compared to these other approaches. Moreover, adsorption has a high treatment efficiency when used to treat both soluble and insoluble pollutants.

## Materials and methods

Stock solutions were initially prepared in n-hexane due to their relatively low water solubility. The extraction of FLU was carried out from n-hexane by the synthesized sorbent, DP. Shaking time, sorbent weight, beginning concentrations, and temperature were all investigated individually as factors impacting FLU sorption. According to Correia et al.^20^ FLU was measured using a UV–visible spectrophotometer at wavelengths of 300 nm (Supplementary Fig. 1S). DP samples were rinsed with distilled water and then dried at 105 °C for 24 h. After cooling, grinding, and sieving to 250 µm were carried out. Physical activation for DP was accomplished by carbonization at temperatures of 500, 700, and 900 °C under N_2_ gas for 2 h, followed by activation using CO_2_ gas for 2 h at 600 °C (Table 1). In fixed-bed column tests, the ideal operating parameters for the aqueous sample were determined to be an inlet flow rate of 10–15 mL min^–1^ for N_2_ gas, an AC concentration of 50 mg L^–1^, and 200 mm.

**Table 1:** Conditions for adsorption parameters.

Conditions	Initial conc mg L^−1^	Removal %		
		DP1	Act. Char. Ref	Raw DP
DP-500 under N_2_ gas for 2 h, then CO_2_ gas for 2 h at 600	24.9	98.9	64.9	25.7
DP-700 under N_2_ gas for 2 h, then CO_2_ gas for 2 h at 600	24.9	75.3	43.8	18.6
DP 900 under N_2_ gas for 2 h, then under CO_2_ gas for 2 h at 600	24.9	55.9	35.3	12.1

The quantity of PAHs that are adsorbing at equilibrium, q_e_ (mg g^−1^) was calculated using the following equation based on mass balance:

1 $${q}_{e }= \frac{v}{m}\left({C}_{o}- {C}_{e}\right)$$

The concentrations of PAHs at the beginning and equilibrium are C_0_ and C_e_, respectively. m is the weight of the adsorbent applied g, and V is the total volume of the solute solution L. The removal percentage of PAHs R% is calculated by using the following formula:

2 $$Removal \%= \frac{{C}_{o}-{C}_{e}}{{C}_{0}} \times 100$$

The distribution coefficient K _d_ is determined from the relationship between the aqueous phase and the solid phase:

3 $${\text{K}}_{\text{d}} = \frac{v}{m}( \frac{{C}_{0 }- {C}_{e}}{{C}_{e}})$$

$${\text{K}}_{\text{d}} = \frac{{q}_{e}}{{C}_{e}}$$

According to the kinetic models, there is a connection between the quantity of PAHs absorbed and the passage of time. In adsorption systems, The kinetic data are described by the Lagergren equation. Furthermore, for pseudo-1st order reactions, assuming both diffusional and surface reaction kinetic models, this equation was created (Eqs. 1–3). The following equation (Eq. 4) gives the pseudo-2^nd^-order kinetic model that describes chemical adsorption:

4 $$\frac{d{q}_{t}}{dt}={k}_{2}{\left({q}_{e}-{q}_{t}\right)}^{2}$$

where *k*_2_ is the rate constant g mg^−1^ min^−1^. Furthermore, an intra-particle diffusion model was investigated to explain the impact of PAH transfer from the solution to the solid surface of the adsorbent during the reaction. The adsorption reaction may be affected by film diffusion, pore diffusion, surface diffusion, and/or adsorption on the pore surface. Because the batch experiment was conducted with shaking, the transfer of adsorbate particles may be characterized by the diffusion coefficient, which fits the experimental results quite well. The thermodynamic parameters corresponding to FLU sorption on the prepared DP were assessed using the Van’t Hoff Eq. (5)

5 $$log{k}_{d}=\frac{\Delta s^\circ }{2.303R}-\frac{\Delta H^\circ }{2.303RT}$$

where *k*_d_ is the distribution coefficient of the solute PAHs, ΔS^◦^ is the entropy change J mol^−1^ K^−1^, *R* is the ideal gas constant 8.314 J mol^−1^ K^−1^ and *T* is the absolute temperature. Arrhenius equation is used to calculate the adsorption process's activation energy as given by Eq. (6).

6 $$ln{k}_{2}=lnA-\frac{{E}_{a}}{RT}$$

where k_2_ g mg^−1^ min^−1^ is the rate constants for the pseudo-2nd-order kinetics, A g mg^−1^ min^−1^ is the temperature-independent Arrhenius factor, Ea k J mol^−1^ is the activation energy, 8.314 J mol^−1^ K^−1^ is the gas constant, and T is the temperature in kelvin.

## Results and discussion

In the FTIR spectrum of FLU/n-Hexane, shown in Supplementary Fig. S2, after using DP physical activation to remove FLU, a new peak appears, referring to FLU at 3265 C^−1^ for C=C–H and another one for C=C at 1651–1696 C^−1^, respectively. which illustrates the DP adsorption accrual. Another peek at 2084 C^−1^ for C–O stretching refers to date pits^21,22^.

### Scanning electron microscopy (SEM)

The microscopic morphological observations of DP before and after the uptake of FLU/n-Hexane are presented in Supplementary Fig. S3A,B. There were large differences between them in surface morphology. The micrograph of DP (Supplementary Fig. S3A) showed a non-uniform complex fiber matrix with no shape. The SEM images of DP produced a highly dense and smooth surface with the absence of pores and the formation of many floccules and spots, leading to a rough appearance. They also displayed small spherical clusters in shape that are strongly agglomerated. On the other hand, in the SEM images of FLU/n-Hexane (Supplementary Fig. S3B), the surface morphology exhibited high porosity compared to DP, revealing macropores and irregular trough-like patterns. The SEM micrographs of FLU/n-Hexane present a sheet-like structure. The SEM photos demonstrate this. The FLU/n-Hexane has a multilayer lamellar layer structure, and the SEM images allow for the identification of individual sheets' borders and the presence of wrinkled regions. It shows that wrinkled and layered flakes were seen on the surface. Shows small spherical particles in shape and strongly agglomerated, and shows spherical clusters made up of fibrous whiskers. Reveal differences in the structure of the surface and the formation of many floccules and spots, leading to a rough appearance, which could be attributed to the adsorption of solutions on the surface of the sorbent material.

### X-ray diffraction (XRD) measurements

The crystalline structure of prepared DP and DP-FLU/n-Hexane was analyzed by XRD in the region of 2θ = 10–80, as shown in Supplementary Fig. S4 The XRD results of the DP show a broad peak that appears between the angles 2θ of 15° to 25°. Moreover, it lacks a horizontal fundamental line, which indicates that the main components of the material are of an amorphous form. The peak widening indicates poor crystallinity. The DP in the XRD profile resembled the DP with FLU/n-Hexane extremely closely.

### Brunauer–Emmett–Teller (BET)

N_2_-BET surface area analysis is used to study material textural properties. Supplementary Figure S5 shows that the adsorption/desorption branch does not close, even at low pressures. To answer the question: is the sample's N_2_ adsorption isotherm correct or incorrect? In theory, the hysteresis at low relative pressure observed in our sample cannot be explained by physisorption. N_2_ at 77K isotherms of materials with mesopores will exhibit hysteresis at p/p° greater than about 0.4, but never at p/p° less than that, at least not significantly lower. The most obvious mechanism/process that causes low-pressure hysteresis is chemisorption. It occurs when some adsorbate is sorbed to high-energy sites' and is difficult to remove. This was proved as before adsorption, the average pore volume of DP was 688.33 cm^3^ g^-1^; following adsorption, it decreased to 310.33 cm^3^ g^-1^. In addition, the relatively high value of Ea = 84.786 demonstrates that the adsorption of FLU onto DP is chemisorption with > 20 kJ mol^−1^. Also, Before adsorption, the surface area of DP was 191.613 m^2^ g^-1^; following adsorption, it was 145.851 m^2^ g^-1^. The pore diameter of DP was 0.300 nm on average. The N_2_-BET surface area data shows that the surface area of DP is decreased, indicating that adsorption took place on the DP surface.

### Thermogravimetric analyses (TGA)

TGA analysis was carried out to test the thermal stability of the prepared material. The TGA of the DP sample illustrates that the sample of DP is pure as was indicated by the absence of any observable weight loss along the heating range of 100–600 °C. Except for very few moistures that were expelled before 100 °C. The TGA of the FLU/DP sample (Supplementary Fig. S6A,B) illustrates that it was loaded with a very low amount of FLU (which evaporates at 295 ºC and was expelled before 300 C followed by no loss of weight.

### Batch investigation

Batch experiments were carried out to determine the best conditions for the synthesized DP to absorb FLU from n-Hexane. The pH, contact time, adsorbent dosage, and adsorbate concentration—adsorption parameters that govern the adsorption process—were optimized.

### Effect of shaking time

Using 0.5 g of the sorbent and a temperature of 25 ± 0.1 °C, the impact of shaking time on the uptake of 1.5 × 10^–4^ M FLU from organic solution was examined in the range of 1–180 min. Supplementary Figure S7 demonstrated that FLU absorption proceeded rapidly with shaking within 10 min, before staying constant, suggesting that contact time was related to the degree of adsorption. In the beginning, there were more binding sites available for FLU adsorption. Because a strong driving force resulted in the quick transfer of adsorbate ions onto the adsorbent's surface, the adsorption process remained constant due to a lack of adsorption sites. With time, the efficiency of absorption decreased. The obtained results indicated that contact time and shaking speed were key parameters for the adsorption process. So, an equilibrium time of 10 min was chosen in future experiments with an uptake of 6.714 mg g^−1^.

### Effect of sorbent weight

In the weight range of 0.05 g for 100 ml of 1.5 × 10^–4^ M FLU solution at 25 ± 1 °C, the results for uptake of the 1 × 10^–4^ M FLU from organic solution utilizing the examined sorbent concerning their weight are shown in Supplementary Fig. S8. The data demonstrated that as the sorbent weight increased from 0.05 to 0.8 g, so did the FLU absorption, which is related to the presence of more surface area, which improved the accessibility of energy sites, which remained consistent as the sorbent weight increased to 1 g. This could be because, with higher sorbent weights, there are more surface functional groups and surface area accessible. In addition, the FLU uptake remained constant at 0.8 to 1 g. When the sorbent dosage is high, this is referred to as sorbent particle aggregation. The overall surface area accessible for FLU sorption is reduced because of this aggregation.

### Effect of sorbent pH

To investigate the effect of pH on the uptake of 1.5 × 10^–4^ FLU from organic solution, 0.5 g of sorbent, 10 min, and 25 ± 1 °C were utilized (Supplementary Fig. S9). The FLU absorption value rose as the pH was raised, when the pH was increased to 6, the absorption dramatically increased. Zwitterions were created when the pH increased because of the OH group's proton donation. This increased the portion of the molecule's susceptibility to transformation. An electron was transferred between the electron-donating group (CO) and the substituent (OH). It is possible that the formation of zwitterions was caused by the molecules' π–π* transitions emerging when the pH increased. As a result, a partial hydrogen bond was formed, with the peak of adsorption occurring at pH 6 and the adsorption remained constant after that. Because FLU molecules are cationic, adsorption is maximized when pH rises because more zwitterions form. For this reason, an equilibrium pH of 6 was used in the next study. This behaviour was associated with the zero net surface charge of the adsorbent, which was determined to be roughly pH 6. Furthermore, because the absorbate and adsorbent repel one another electrostatically when the pH drops below 6, the adsorbent's surface becomes positively charged, inhibiting FLU adsorption. Furthermore, H + ions compete with the function groups at the FLU at low pH values. Therefore, the pH level may have a major effect on the adsorbent's activity as well as the absorbent's functional groups. At high pH of the contact solution, adsorption occurs by ion exchange while at low pH, the process is physical. Based on free energy change, we can conclude that the process occurs spontaneously and is more pronounced at low acidity. As the pH decreased, the elimination efficiency increased with the use of all sorbents. Therefore, we can generally state that in zwitterions, substances behave as bases with decreasing pH. They are strong acceptors of π-electrons.

### Effect of initial concentrations of FLU

By altering the beginning FLU organic solution concentrations from 1.5 × 10^–4^ to 0.1 × 10^-5^ M, the effect of initial FLU organic solution concentrations on absorption onto the studied sorbent was investigated. The outcomes are given in Supplementary Fig. S10. It was revealed that as the initial DP concentration increased, the uptake of FLU absorption increased. The increase in absorption could be attributable to a rise in the concentration gradient's driving force. This behavior was predicted due to the presence of a site on the adsorbent, which served as a stumbling block to FLU elimination.

### Effect of temperature

The effect of temperature on the uptake of 1.5 × 10^–4^ M FLU from organic solution was examined with 0.1 g of sorbent at various temperatures within the range of 25–55 °C. From the results, it is found that FLU uptake decreased somehow with rising temperature, according to the findings (Supplementary Fig. S11). This data indicates that the FLU uptake process via organic solution-prepared sorbent is exothermic. Lowered FLU mobility and a reduction in the retarding forces acting on the diffusing FLU may be the causes of the decline in FLU uptake at the high temperature utilizing DP. For determining the adsorption mechanism, the amount of FLU adsorption onto 0.5 g of prepared DP was investigated as a function of time. Supplementary Figure S12 showed that a mixing period of 10 min was the best condition. This result indicated that the FLU adsorbs quickly onto the prepared sorbent. The obtained data were subjected to various kinetic models, and the kinetic parameters were calculated. The adsorbed amount of FLU is correlated with time in different models. For pseudo 1st-order reactions, the Lagergren equation was used Eqs. (1–4). The k_1_ was found by plotting log (q_e_–q_t_) with t, and the value of q_e_ was found by looking at the intercept. The kinetic parameters were determined in the given Table 2. Linear fit with correlation coefficients R^2^ = 0.082, as seen in Supplementary Fig. S13. for adsorption capacity qe, and R^2^ clarified that the studied kinetic model did not fit well with the experimental results (6.714 mg g^−1^).

**Table 2:** Kinetic model parameters for adsorption of FLU onto the synthesized sorbent.

Adsorption	Kinetic model	Parameters	R^2^
FLU/DP	Pseudo-1st order at 25 °C	k_1_ = 0.00898 and q_e_ = 1.645	0.082
	Pseudo-2nd order at 25 °C	k_2_ = 0.0258 and q_e_ = 3.7271	0.991
	Pseudo-2nd order at 30 °C	k_2_ = 0.0501 and q_e_ = 2.6205	0.997
	Pseudo-2nd order at 35 °C	k_2_ = 0.3260 and q_e_ = 1.1284	0.999
	Pseudo-2nd order at 40 °C	k_2_ = 0.3611 and q_e_ = 0.9546	0.999
	Pseudo-2nd order at 45 °C	k_2_ = 0.6579 and q_e_ = 0.7487	0.999
	Pseudo-2nd order at 50 °C	k_2_ = 0.6083 and q_e_ = 0.5910	0.997
	Pseudo-2nd order at 55 °C	k_2_ = 0.4696 and q_e_ = 0.6651	0.999
	Intra particle	kid = 0.0144 and C = 3.5776	0.773

The 2nd order kinetic model, which describes chemical adsorption is given by Ho and McKay^23^. The model parameters were calculated from the plot of t/qt with t as displayed in Supplementary Fig. S14. The plot showed linear relationships, and the model parameters and R^2^ are given in Table 3. The results demonstrated better linear fitting to the pseudo-2nd-order kinetic model. This finding referred to the participation of chemical performance of the adsorption of the FLU onto the DP.

**Table 3:** Adsorption isotherm models parameters for the sorption of FLU onto the synthesized DP.

Adsorption system	Isotherm model	Parameters	R^2^
FLU/DP	Langmuir	*q*_*max*_ = 0.666 *K*_*L*_ = 0.0833	0.930
	Freundlich	*K*_*f*_ = 0.077 $\frac{1}{n}$= 1.519	0.942

The Weber model; the Intra-particle diffusion model was also studied to explain the influence of the transfer of FLU from organic solution to the solid surface of adsorbent at the reaction. The adsorption reaction could be affected by film diffusion, pore diffusion, surface diffusion, and/or adsorption on the pore surface. The studied batch experiment was performed with shaking; therefore, the transfer of adsorbate particles could be described by diffusion coefficient. Weber model was applied as shown by Equation below:

$${q}_{t}= {k}_{id}{t}^{0.5}+ C$$

where *k*_id_ is the Weber model constant mg g^−1^ min^−0.5^ and *C* is a constant mg g^−1^ connected to the depth of the boundary layer, which reflects the boundary layer effect. If the adsorption takes place within a multilayer adsorbent, the adsorbate particles must spread within the interior pores of solid materials. The model parameters were obtained from the plot of *q*_t_ vs. *t*^1/2^ as shown in Supplementary Fig. S14. The results showed two linear regions referring to the participation of at least two steps in the reaction. The linearity in the first region referred to a diffusion of FLU into macro-pores of adsorbent, while the second linear region showed that the adsorbate particles diffused within a micro-pore of adsorbent. The obtained results reflected a variation in particle migration rate between different stages of sorption.

### Adsorption isotherm

Various isotherm models were studied to explain the adsorption mechanism regulating the reaction. In this work, the results of the experiment were obtained using Langmuir isotherm model for the adsorption equilibrium of FLU onto the AC which are expressed as:

$$\frac{1}{{q}_{e}}=(\frac{1}{{K}_{a}{q}_{m}})\frac{1}{{C}_{e}}+\frac{1}{{q}_{m}} $$

where *C*_e_ is the PAHs concentration in solution after experiment mg/L, *q*_e_ is PAHs concentration on the solid DP mg/g, and *q*_max_ and *K*_L_ are the model parameters connected to the maximum adsorbed amount mg/g and adsorption energy, respectively. A plot of 1/*C*_e_ vs. *1*/*q*_e_ is presented in Supplementary Fig. S15 and the model parameters were determined from the plot and given with the correlation coefficient R^2^ (Table 3). The value of R^2^ for the adsorption system was found to be 0.930. This value reflected a fair fit with the Langmuir isotherm. Moreover, as compared to the obtained experimental data, the uptake value is not in agreement with the calculated value obtained from Langmuir.

### Freundlich isotherm model

It was applied to the experimental results, described by Eqs. (1)–(2) as monitored previously: where k_f_ mg g^−1^ and n are the model constants, indicating the adsorption capacity and favorability nature of the adsorption process, respectively. Freundlich model constants were determined from the linear fit of the log q_e_ vs. log C_e_ plot as shown in Supplementary Fig. S16, and the results are given in Table 3. The value of R^2^ of the Freundlich plot (0.942) showed good fits of the experimental results with the Freundlich isotherm model. This finding proved the participation of the multilayer chemical adsorption process onto heterogeneous surfaces.

### Adsorption thermodynamics

ΔG, ΔH, and ΔS were estimated using the Van’t Hoff equation. The − ΔG confirmed the spontaneous nature of the adsorption process. The values of ΔH and ΔS were obtained from the slope and intercept of the plot of log K_d_ versus 1/T as illustrated in Supplementary Fig. S17 and (Table 4) The − ΔH demonstrated the exothermic nature of the adsorption process. The + ΔS corresponds to an increase in the degree of freedom of the adsorbed FLU. In addition, the positive value of ΔS reflects the affinity of the adsorbent towards the adsorbate molecules. It also suggests more randomness at the interface between the solid and the solution.

**Table 4:** Thermodynamic parameters for the adsorption of FLU using the synthesized sorbent.

Adsorption system	Tem. K	∆*H*° (k J mol ^−1^)	∆*G*° (kJ mol^−1^)	∆*s*° (J K^−1^ mol^−1^)
FLU/DP	298	− 5.744E−6	− 0.0205	0.069
	303		− 0.0209	
	308		− 0.0212	
	313		− 0.0216	
	318		− 0.0219	
	323		− 0.0222	
	328		− 0.0226	

### Mechanism of adsorption

The adsorption equilibrium results suit the Freundlich model perfectly. This finding proved the participation of the multilayer chemical adsorption process on heterogeneous surfaces of DP. The kinetic adsorption data were well fitted to the pseudo-2nd order model related to the contribution of chemical adsorption for the FLU onto the synthesized DP (Supplementary Figs. S18–S19). As previously mentioned, the Ea value indicates whether the adsorption is a physical or chemical process. The relatively high value of Ea = 84.786 (Table 5) demonstrates that the adsorption of FLU onto DP is chemisorption with > 20 kJ/mol^23^. The capacity of DP for adsorbing of FLU is higher compared to other adsorbents (Table 6).

**Table 5:** Activation energy and rate constants of pseudo-2nd-order kinetics for the adsorption of FLU onto DP.

Temp. ℃	K_2_ (g mg^−1^ min^−1^)	Ea (kJ mol^−1^)	R^2^
25 °C	0.0258	84.786	0.9907
30 °C	0.0501		0.9967
35 °C	0.3260		0.9989
40 °C	0.3611		0.9991
45 °C	0.6579		0.9993
50 °C	0.6083		0.9971
55 °C	0.4696		0.9988

**Table 6:** Comparison between different capacities of some adsorbent materials for the removal of FLU.

Adsorbents	PAHs	Adsorption capacity mg g^−1^	References
DPPA	FLU	6.714	Present work
ENPM	PAHs	0.43 ± 0.045	^24^
SG	FLU ACY	2.12 1.13	^25^
AdMC	ACE FLU ANT PHE	0.4970 0.4997 0.4902 0.5014	^26^
GAC	FLU	13.2	^27^
AC	NAP FLU	13.57 12.95	^28^
NSMC	PHE ACY FLU	2.50 1.20 0.90	^29^

*ACE* Acenaphthene, *ACY* Acenaphthylene, *AC* Activated carbon, *ANT* Anthracene, *AdMC* asphalt-derived mesoporous carbon, *ENPM* Electrospun nanofibrous polycyclodextrin membranes, *GAC* Granular activated carbon, *DPAA* Date pits physical activation, *NAP* Naphthalene, *NSMC* Natural, synthetic, and modified clays, *PHE* Phenanthrene, *SG* Seagrass powder.

### Desorption of FLU

The natural Saudi volcanic ash's remarkable regeneration ability following multiple cycles of desorption and adsorption suggests that it has a great deal of potential as an excellent adsorbent for the removal of dyes from aqueous systems^24^. Reusability experiments involved using ethanol, sodium hydroxide, acetic acid, hydrochloric acid, and sulfuric acid to deplete the regenerated DP (Table 7). From the data collected, it was discovered that ethanol is the best, even after the fourth cycle, with a maximum regeneration efficiency of 86%. Contrarily, for sodium hydroxide, acetic acid, hydrochloric acid, and sulfuric acid, it was 12, 38, 22, and 34%, respectively. X-ray diffraction was used to calculate the morphology for each cycle. The characteristic peak for DP loaded with FLU was obtained after the fourth cycle of deformation. This may be because physical regeneration involves the expansion of the DP-AC pores in each cycle, which may affect the characteristics of the adsorbent after repeated cycles, leading to a drop in regeneration efficiency.

**Table 7:** Regeneration efficiency using different organic solvents.

Organic solvent	Efficiency %
Ethanol	86
Sodium hydroxide	12
Acetic acid	38
Hydrochloric acid	22
Sulphuric acid	34

### Feasibility study

The gases used to produce 1 g of DP from date seeds are CO_2_ and N_2_ gas which have a total cost of 30 SR. And the cost of electricity consumption has reached 1.4 SR. (4.5ℎr × 1.8 kw × 0.18 *SR*). At the same time, the cost of tap water was 5 SR. The cost of human resources was 80 SR (2 persons × 40 *SR*) for 48 h. Thus, the total cost of producing 1 g of DP in this study was 115.4 SR. Also, the result of XRD shows we can reuse the prepared DP without significantly decreasing performance for 4 times. From all this, it appears to us that AC prepared from DP is a promising adsorbent with a low cost for the removal of many organic pollutants.

## Conclusion

It was possible to successfully prepare waste-derived AC from DP as an efficient and environmentally friendly biosorbent for the removal of FLU from aqueous solutions. The maximaum DP capacity of 6.714 mg g^−1^ was found to agree with the Freundlich model. A superior linear fit for the pseudo-2nd-kinetic model was followed by FLU adsorption's chemisorption performance on DP. The maximum adsorption capacity (${q}_{e}$) from the pseudo-2nd order kinetic model fitted with the experimental findings and found to be 3.727 g, 2.621, 1.128, 0.955, 0.749, 0.591, and 0.665 mg g^−1^ at 25, 30, 35, 40, 45, 50 and 55 °C, respectively. A negative value for ∆G indicates that the adsorption was spontaneous, consistent with an exothermic nature. On the other hand, a positive value for + ΔS indicates an increase in the degree of freedom for FLU adsorption. The adsorption of FLU onto DP is categorized as chemical adsorption due to the comparatively high activation energy (Ea), which is 84.786 kJ mol^−1^. Additionally, the XRD result demonstrates that the prepared DP can be reused four times without noticeably lowering performance. Furthermore, it seems that AC made from DP is a cheap and effective adsorbent for eliminating a variety of organic contaminants.

## Supplementary Information

Supplementary Information.

## Data Availability

All required data will be available with the corresponding author upon request.

## References

[1] Alegbeleye, O. O., Opeolu, B. O. & Jackson, V. Bioremediation of polycyclic aromatic hydrocarbon (PAH) compounds: (acenaphthene and fluorene) in water using indigenous bacterial species isolated from the Diep and Plankenburg rivers, Western Cape, South Africa. *Braz. J. Microbiol.* **48**, 314–325 (2017). 27956015 10.1016/j.bjm.2016.07.027 PMC5470342

[2] Kuppusamy, S. *et al.* Remediation approaches for polycyclic aromatic hydrocarbons (PAHs) contaminated soils: Technological constraints, emerging trends, and future directions. *Chemosphere* **168**, 944–968 (2017). 27823779 10.1016/j.chemosphere.2016.10.115

[3] Wang, Z. *et al.* Large-scale fabrication of N-doped porous carbon nanosheets for dye adsorption and supercapacitor applications. *Nanoscale* **11**(18), 8785–8797 (2019). 31032826 10.1039/c9nr01777a

[4] Naji, S. Z. & Tye, C. T. A review of the synthesis of activated carbon for biodiesel production: Precursor, preparation, and modification. *Energy Convers. Manag. X* **13**, 100152 (2022).

[5] Surkatti, R., Ibrahim, M. H. & El-Naas, M. H. Chapter 7-Date pits activated carbon as an effective adsorbent for water treatment. In *Sorbents Materials for Controlling Environmental Pollution *(ed. Avelino, N.-D.) 135–161 (Elsevier, 2021). 10.1016/B978-0-12-820042-1.00007-9.

[6] Reza, M. S. *et al.* Preparation of activated carbon from biomass and its applications in water and gas purification, a review. *Arab J. Basic Appl. Sci.* **27**(1), 208–238. 10.1080/25765299.2020.1766799 (2020).

[7] Heidarinejad, Z. *et al.* Methods for preparation and activation of activated carbon: A review. *Environ. Chem. Lett.* **18**, 393–415. 10.1007/s10311-019-00955-0 (2020).

[8] El-Naas, M., Sulaiman, A. & Abu Alhaija, M. Removal of phenol from petroleum refinery wastewater through adsorption on date-pit activated carbon. *Chem. Eng. J.* **162**(3), 997–1005. 10.1016/j.cej.2010.07.007 (2010).

[9] Eeshwarasinghe, D. *et al.* Removing polycyclic aromatic hydrocarbons from water using granular activated carbon: Kinetic and equilibrium adsorption studies. *Environ. Sci. Pollut. Res.* **25**(14), 13511–13524. 10.1007/s11356-018-1518-0 (2018). 10.1007/s11356-018-1518-0 29492819

[10] Kumar, J. A. *et al.* Enhanced PAHs removal using pyrolysis-assisted potassium hydroxide induced palm shell activated carbon: Batch and column investigation. *J. Mol. Liq.* **279**, 77–87. 10.1016/j.molliq.2019.01.121 (2019).

[11] Gupta, H. & Gupta, B. Adsorption of polycyclic aromatic hydrocarbons on banana peel activated carbon. *Desalin. Water Treat.* **57**(20), 9498–9509 (2016).

[12] Nor El Houda, M., Chabani, M., Bouafia-Chergui, S. & Touil, A. Removal of chemical oxygen demand from real petroleum refinery wastewater through a hybrid approach: Electrocoagulation and adsorption. *J. Chem. Eng. Process. Process Intensif.* **196**, 109680. 10.1016/j.cep.2024.109680 (2024).

[13] Alhusnawy, Z. J. & Alsultani, K. F. Enhancing toluene adsorption on ZnCl_2_ one-step modified corn cob activated carbon. *Ecol. Eng. Environ. Technol.* **25**(6), 104–114. 10.12912/27197050/186669 (2024).

[14] Khalaf, H. K. & Rashid, H. M. Dates pits activated carbon as cheap sorbent for the decontamination of the cadmium ions in battery mills wastewater. *Iraqi J. Chem. Pet. Eng.* **25**(2), 119–129 (2024).

[15] Lei, Y. *et al.* Biomimetic ZrO_2_-modified seaweed residue with excellent fluorine/bacteria removal and uranium extraction properties for wastewater purification. *Water Res.* **252**, 121219. 10.1016/j.watres (2024). 38309067 10.1016/j.watres.2024.121219

[16] Guo, W. *et al.* Efficient removal of fluorine by carbon fiber supported Mg-Fe binary metal oxide composite adsorbent and mechanism analysis based on DFT. *Sep. Purif. Technol.* **330**(3), 125320. 10.1016/j.seppur.2023.125320 (2024).

[17] Alharbi, H. A., Hameed, B. H., Alotaibi, K. D., Al-Oud, S. S. & Al-Modaihsh, A. S. Recent methods in the production of activated carbon from date palm residues for the adsorption of textile dyes: A review. *Front. Environ. Sci.*10.3389/fenvs.2022.996953 (2022).

[18] Hua, L., Wu, W., Liu, Y., Chen, Y. & McBride, M. B. Effect of composting on polycyclic aromatic hydrocarbons removal in sewage sludge. *Water Air Soil Pollut.* **193**(1), 259–267 (2008).

[19] Al-Zuhair, S., Noura, H. & Fardoun, A. Using activated carbon from waste date-pits as an adsorbent for transformer oil regeneration. In *World Congress on Sustainable Technologies (WCST)*, London, UK 69–72. 10.1109/WCST19361.2011.6114241 (2011).

[20] Correia, F. C., Santos, T. C. F., Garcia, J. R., Peres, L. O. & Wang, S. H. Synthesis and characterization of a new semiconductor oligomer having quinoline and fluorene units. *J. Braz. Chem. Soc.* **26**, 84–91 (2015).

[21] Liu, Y. L., Chang, C. Y., Hsu, C. Y. & Chou, I. C. Preparation, characterization, and properties of fluorene-containing benzoxazine and its corresponding cross-linked polymer. *J. Polym. Sci. Part A Polym. Chem.* **48**(18), 4020–4026 (2010).

[22] Al-Swaidan, Hassan, M. & Ashfaq, A. Synthesis and characterization of activated carbon from Saudi Arabian dates tree’s fronds wastes. In *3rd**International Conference on Chemical, Biological and Environmental Engineering*, vol. 20, 25–31 (2011).

[23] Ho, Y. S. & McKay, G. The kinetics of sorption of basic dyes from aqueous solution by sphagnum moss peat. *Can. J. Chem. Eng.* **76**(4), 822–827 (1998).

[24] Celebioglu, A., Topuz, F., Yildiz, Z. I. & Uyar, T. Efficient removal of polycyclic aromatic hydrocarbons and heavy metals from water by electrospun nanofibrous polycyclodextrin membranes. *Am. Chem. Soc. Omega* **4**(4), 7850–7860 (2019). 10.1021/acsomega.9b00279 PMC6648243 31459873

[25] Akinpelu, A. A., Nazal, M. K. & Abuzaid, N. Adsorptive removal of polycyclic aromatic hydrocarbons from contaminated water by biomass from dead leaves of Halodule uninervis: Kinetic and thermodynamic studies. *Biomass Convers. Bioref.* **13**, 8301–8313 (2023).

[26] Alissa, F. M. *et al.* Synthesis of highly efficient asphalt-based carbon for adsorption of polycyclic aromatic hydrocarbons and diesel from emulsified aqueous phase. *Carbon Lett.* **30**(5), 555–567 (2020).

[27] Awoyemi, A. Understanding the adsorption of PAHs from Aqueous Phase onto Activated Carbon. *MASc thesis*, University of Toronto Toronto, Canada (2011).

[28] Satouh, S. *et al.* Adsorption of polycyclic aromatic hydrocarbons by natural, synthetic and modified clays. *Environments* **8**(11), 124 (2021).

[29] Muazu, N. D. *et al.* Volcanic ash and its NaOH modified adsorbent for superb cationic dye uptake from water: Statistical evaluation, optimization, and mechanistic studies. *Colloids Surf. A Physicochem. Eng. Asp.* **634**, 127879 (2022).

