# Supplementary material for: Biosorbent treatment of fluorene using activated carbon derived from the pyrolysis process of date pit wastes
Source: Sci Rep. 2024 Sep 26;14:22039. doi: 10.1038/s41598-024-72127-2 (PMC11427576; doi:10.1038/s41598-024-72127-2)
Supplement: Supplementary file 1 — Supplementary Information. [file 41598_2024_72127_MOESM1_ESM.zip › Supplementary file 27 August 2024.docx]

**Biosorbent Treatment Of Fluorene Using Activated Carbon Derived From The Pyrolysis Process Of Date Pit Wastes**

**Tarek O. Said^*1^, Badriah S. Al-Farhan^2^, Sara A. El-Ghamdi^2^, Nasser Awwad^3^**

**Figure** **1S.** UV/V Spectrophotometric analysis of FLU.

**
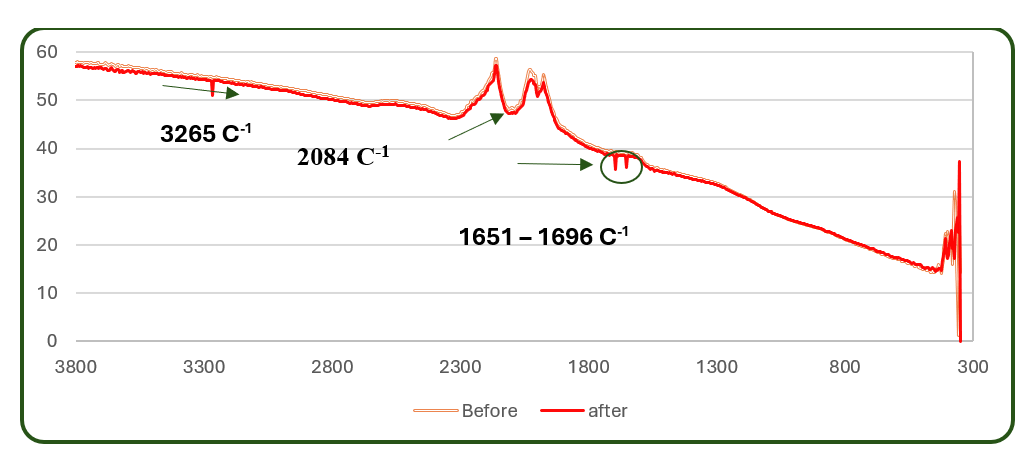
**

**Figure 2S.** FTIR of DP and FLU/n-Hexane.


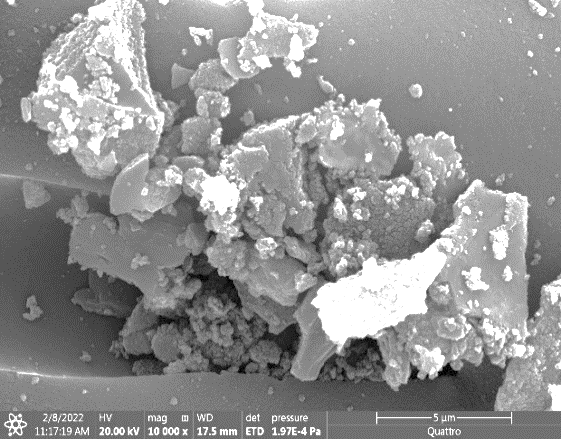

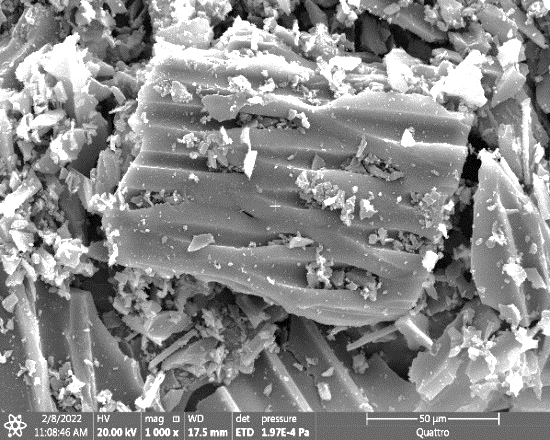

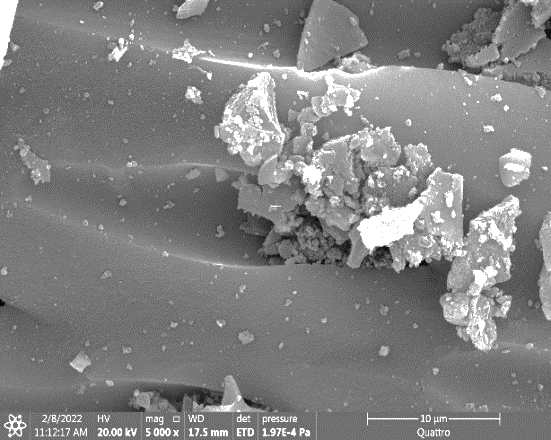

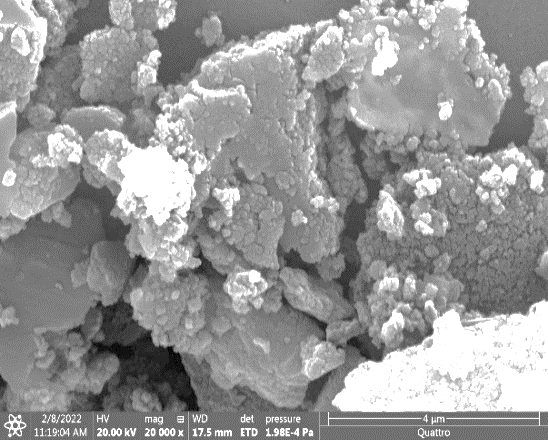
**Figure 3S A.** SEM images of DP before adsorption.

**
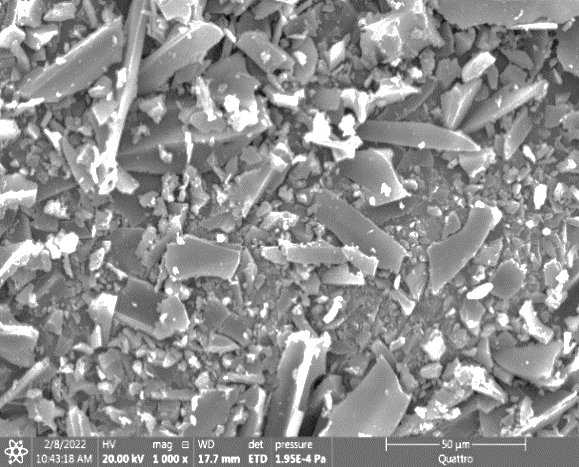

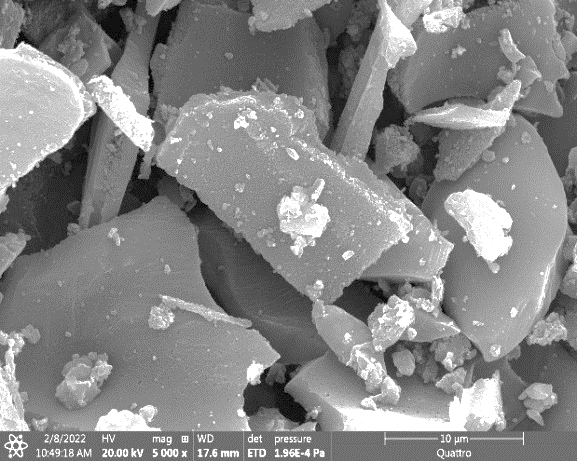

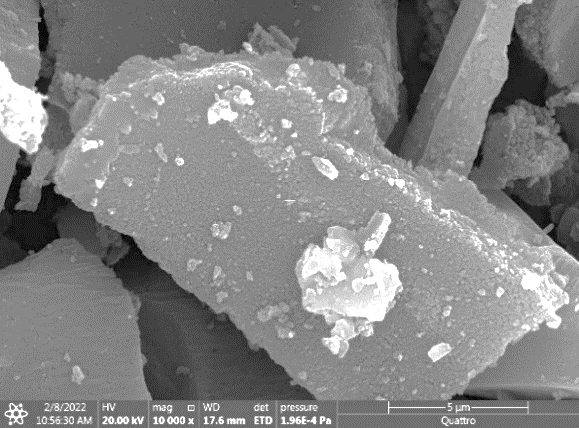

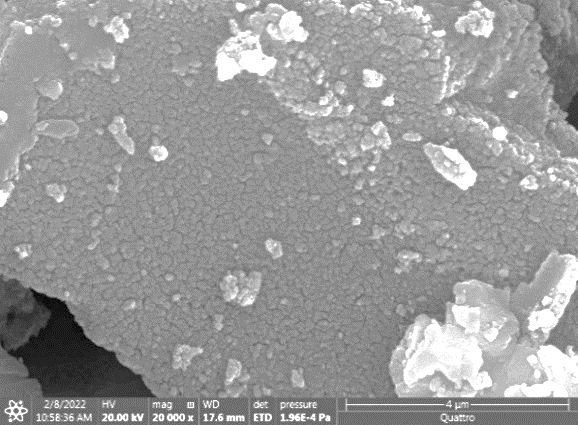
**

**Figure 3S B.** SEM images of DP after adsorption.

**Figure 4S.** XRD patterns of DP and DP with FLU/n-Hexane.

**Figure 5S.** N_2_-BET of DP before and after adsorption.

| 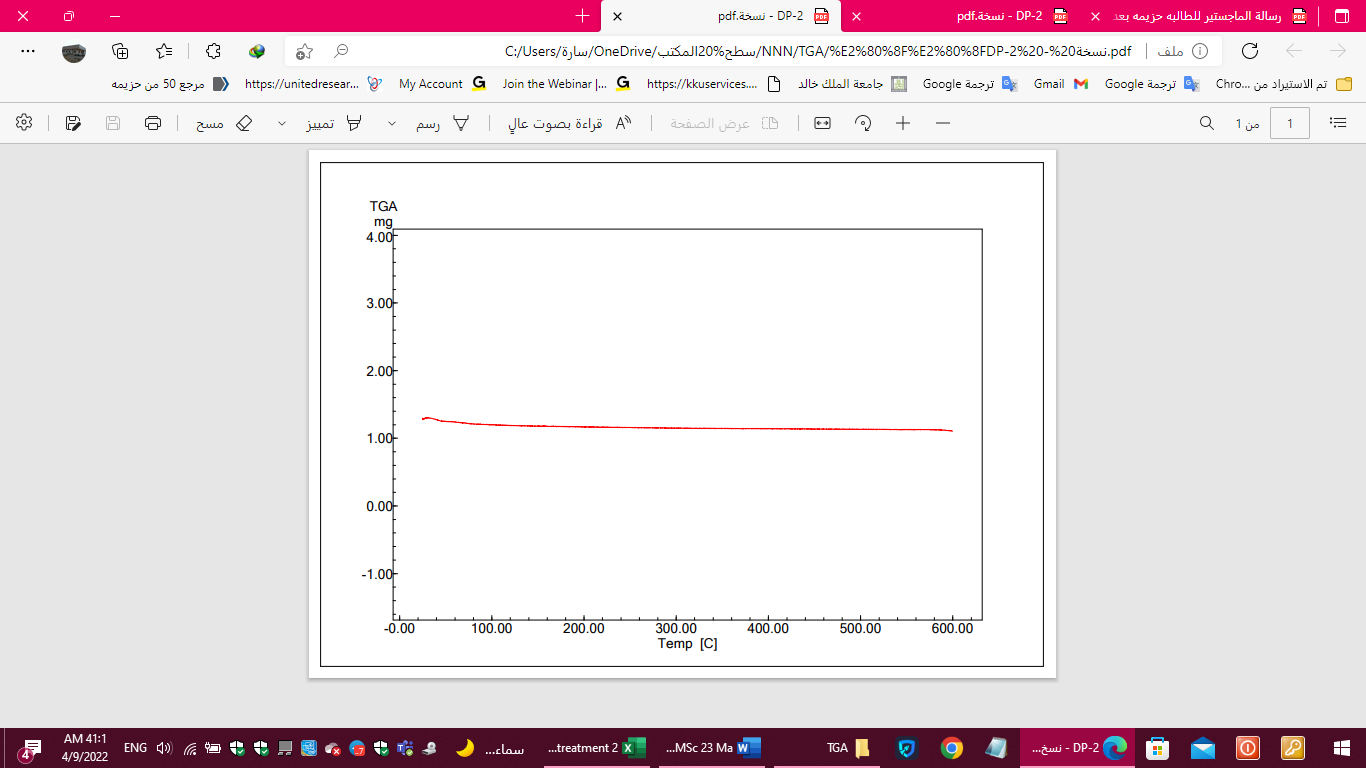  A | 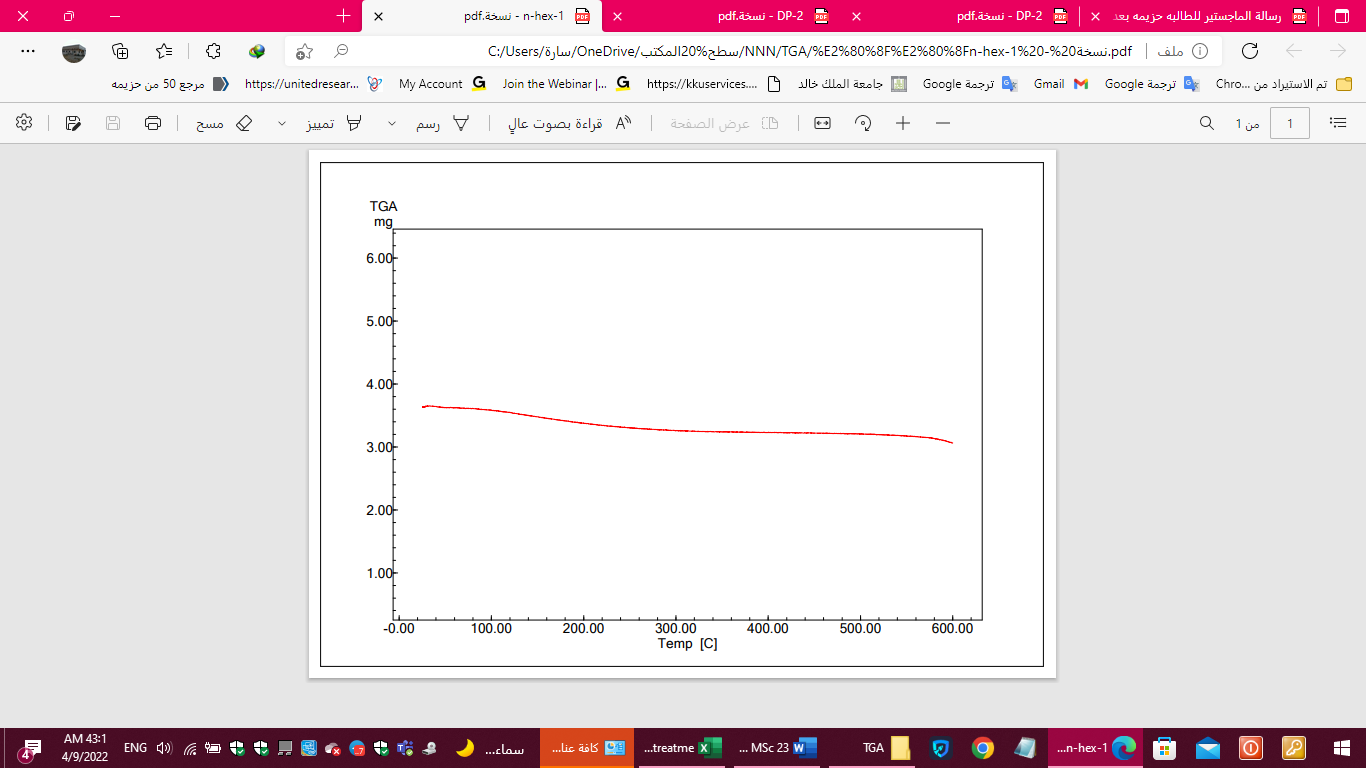  B |
| --- | --- |
| **Figure 6S A** TGA of DP: A) before adsorption and (B) after adsorption. | |

**Figure 7S.** Effect of shaking time on the uptake of FLU from n-hexane onto synthesized DP.

**Figure 8S.** Effect of DP dosage on the uptake of FLU from n-hexane onto synthesized DP.

**Figure 9S.** Effect of pH on the uptake of FLU from n-hexane onto synthesized DP.

**Figure 10S.** Effect of concentration on the uptake of FLU from n-hexane onto synthesized DP.

**Figure 11S** Effect of temperature on the uptake of FLU from n-hexane onto synthesized DP.

**Figure 12S** Pseudo1^st^ - order model kinetic plot for the sorption of FLU onto the synthesized DP.

**Figure 13S.** Pseudo- 2nd-order model kinetic plot for the sorption of FLU onto the synthesized DP.

**Figure 14S.** Intra-particle kinetic model plot for the sorption of FLU onto the synthesized DP.

**Figure 15S.** Langmuir isotherm plot for the uptake of FLU onto the synthesized DP.

**Figure 16S.** Freundlich model isotherm plot for the uptake of FLU onto the synthesized DP.

**Figure 17S.** Effect of temperature on the sorption of FLU from n-hexane onto synthesized DP.

**Figure 18S** Pseudo-2^nd^-order model kinetic plot for the sorption of FLU onto the synthesized DP.

**Figure 19S.** Arrhenius plot for the uptake of FLU onto DP.
